# Antidepressants as Endocrine Disrupting Compounds in Fish

**DOI:** 10.3389/fendo.2022.895064

**Published:** 2022-06-16

**Authors:** William Andrew Thompson, Mathilakath M. Vijayan

**Affiliations:** Department of Biological Sciences, University of Calgary, Calgary, AB, Canada

**Keywords:** hormones, reproduction, growth, stress response, monoamines, steroids, pharmaceutical, municipal wastewater effluent

## Abstract

As antidepressant usage by the global population continues to increase, their persistent detection in aquatic habitats from municipal wastewater effluent release has led to concerns of possible impacts on non-target organisms, including fish. These pharmaceuticals have been marketed as mood-altering drugs, specifically targeting the monoaminergic signaling in the brain of humans. However, the monoaminergic systems are highly conserved and involved in the modulation of a multitude of endocrine functions in vertebrates. While most studies exploring possible impact of antidepressants on fish have focused on behavioural perturbations, a smaller spotlight has been placed on the endocrine functions, especially related to reproduction, growth, and the stress response. The purpose of this review is to highlight the possible role of antidepressants as endocrine disruptors in fish. While studies linking the effects of environmentally relevant levels of antidepressant on the endocrine system in fish are sparse, the emerging evidence suggests that early-life exposure to these compounds have the potential to alter the developmental programming of the endocrine system, which could persist as long-term and multigenerational effects in teleosts.

## Introduction

Antidepressants in aquatic environments have persisted for decades and are continually introduced into our waterways through municipal wastewater effluent (MWWE) release (1). Aquatic organisms, including fish, inhabiting these waterways are persistently exposed to these compounds, but effects on non-target organisms are only beginning to emerge (2, 3). Over the last few decades, studies have examined the effects of antidepressants on teleosts, but the majority of work have focused on behavioural alterations (4–6). Monoamines, including serotonin, noradrenaline, and dopamine, are the main targets by which the antidepressants elicit a behavioural response (7). However, monoamines play significant roles in modulating other endocrine functions, including steroid hormone synthesis and action (8–13), suggesting wider impact on the physiology of fishes due to antidepressant exposures. As recent reviews have discussed the impact of antidepressants on behavioral consequences (5, 6), this minireview will be limited to the endocrine-related perturbations in fish. Here, we highlight the emerging evidence of antidepressants as endocrine disrupting compounds in fish.

## Antidepressants in the Aquatic Environment

Since the use of anti-histaminergic compounds in the early 1950’s, the practice of neuroactive compounds to treat behavioural deficits associated with major depressive disorder has grown (14–16). The advent of selective serotonin reuptake inhibitors (SSRI) reshaped the antidepressant market, and prescriptions increased rapidly in the early 1990s, especially with the release of fluoxetine (Prozac), citalopram (Celexa), fluvoxamine (Luvox), paroxetine (Paxil), and sertraline (Zoloft; 17). To diminish the side-effects associated with the SSRI class of compounds, additional antidepressants targeting the inhibition of reuptake of either noradrenaline (NRI), such as reboxetine (Edronax), or both serotonin and noradrenaline (SNRI), such as venlafaxine (Effexor) and duloxetine (Cymbalta), emerged (17). In humans, antidepressants typically undergo first-pass metabolism in the liver (18), and in many cases their metabolites, including norfluoxetine (fluoxetine), O-desmethylvenlafaxine (venlafaxine), and desmethylcitalopram (citalopram), remain equipotent to the parent compound (19). Consequently, excretion of these drugs, and the incomplete removal by wastewater treatment processes, has led to the antidepressants, including citalopram, sertraline, venlafaxine, fluoxetine, and other neuroactive compounds, being prevalent in MWWE at concentrations greater than parts per billion (20–27). Also, given their continued release into the environment and their slower breakdown, antidepressants are being touted as persistent contaminants in the aquatic environment (28). Given that antidepressants consumption has gone up in the last 20 years and is only expected to increase (29, 30), environmental levels will also increase in our waterways receiving MWWE outfalls. Consequently, understanding the impact of these drugs on non-target animals, such as fishes, and deciphering their mode of action leading to the adverse effects, will allow for developing environmental risk assessment models for antidepressants in our waterways.

## Antidepressant Effects on Monoamine Content in Fish

In humans, antidepressants primarily target the transporters of monoamines, including serotonin, noradrenaline, and dopamine, inhibiting the reuptake of monoamines from the synaptic cleft into the presynaptic cell (6, 31). This increases the duration of exposure, as well as the concentration of monoamines in the synapse, leading to increased interaction with receptors on the postsynaptic terminal for therapeutic benefits (31, 32). In teleosts, studies have demonstrated that antidepressants interact with the monoamine transporters, and disrupt brain monoamines content (33–41). For instance, both fluoxetine and venlafaxine reduced brain serotonin levels in adult hybrid striped bass (*Morone saxatilis*; 33, 37, 38) and Siamese fighting fish (*Betta splendens*; 35). Also, exposures to venlafaxine elevated serotonin, noradrenaline, and dopamine contents in a region-specific manner in the midbrain of juvenile rainbow trout (*Oncorhynchus mykiss;* 36). Early developmental exposure to venlafaxine depleted serotonin immunoreactivity in the larval zebrafish (*Danio rerio*) brain and reduced catecholamine cell populations (39). These results suggest that antidepressants impact on brain monoamine content in fishes may be species- and/or life-stage-specific (6, 13), but a clear cause and effect relationship has not been established.

## Antidepressants Effect on the Endocrine Systems in Fish

The monoaminergic system is highly conserved in vertebrates, and the central and peripheral monoamines play an important role in modulating endocrine functions (3, 8–10, 12, 42–49). Consequently, disruptions in the monoaminergic pathway is a potential target for antidepressants effect on the endocrine system (6, 13). Very little is known about the mode of action of antidepressants in disrupting the endocrine axes in teleosts. Here we will outline some of the studies that suggest that the antidepressants are endocrine disruptors, especially pertaining to reproduction, growth, and the stress response in fish.

### Reproduction

Reproduction is primarily under the control of the hypothalamic-pituitary-gonadal (HPG) axis in fish (9, 50). The hypothalamus releases gonadotropin releasing hormone (GnRH), the primary endocrine signal for the release of the pituitary gonadotropins, including the follicular stimulating hormone (FSH) and luteinizing hormone (LH), into the circulation (50, 51). The actions of LH and FSH stimulates the secretion of the sex steroids, including androgens and estrogens/progestins, essential for spermatogenesis and oogenesis, respectively (52). There is evidence that antidepressants, both SSRI’s and the SNRI’s, affect reproductive performance in fish (see **Table 1** for specific experimental information). For instance, fluoxetine exposure for 100 d delayed the onset of sexual maturation in the male mosquitofish (*Gambusia affinis*; 53). Also, fluoxetine exposure for over 7 d increased estrogen level, while reducing testosterone level and the milt volume in the male goldfish (*Carassius auratus*; 55). Citalopram exposure reduced whole-brain GnRH transcripts and disrupted spermatogenesis in male zebrafish (60). In females, fluoxetine exposure reduced estradiol levels in the ovary, and this correlated with a drop in egg production in zebrafish (56). A similar result was also seen in goldfish, with fluoxetine injection leading to significant reductions in circulating estradiol levels (54). Venlafaxine exposure also reduced egg production in adult zebrafish (78). Although these studies suggest that both the SSRI and the SNRI class of antidepressants have the potential to affect fish reproduction, the concentrations utilized in these studies were high and not environmentally realistic. Few studies have assessed reproductive parameters in fish upon exposure to environmentally relevant concentrations. For instance, fluoxetine (0.1 ug/L) exposure for 4 weeks perturbed estradiol levels, but did not affect the reproductive performance in the female Japanese medaka (*Oryzias latipes*; 59). Also, exposures to environmentally relevant concentrations of fluoxetine (30 or 380 ng/L) increased total sperm count (58), but did not affect sperm characteristics (79) in the Eastern mosquitofish (*Gambusia holbrooki*). These studies suggest that although exposure of adult fish to environmentally relevant levels may cause endocrine disruption, it did not appear to pose *a negative* risk to the reproductive outcomes. Life stage of exposure must also be considered, as growing evidence from recent studies reveal that early-life stages may be particularly sensitive to antidepressants exposure and may lead to longer-term reproductive impairment. For instance, life-long exposure to fluoxetine (0.5 or 5 µg/L) in turquoise killifish (*Nothobranchius fuzeri*) increased fecundity (57). Also, venlafaxine over the entire life cycle at concentrations as low as 0.88 µg/L reduced the length of the ovipositor in female fathead minnows (*Pimephales promelas*), while at 88 µg/L there was an increase in the total egg output of females (61). These latter studies highlight two critical lessons from contaminant studies: i) species residing in aquatic habitats receiving MWWE may be sensitive to these compounds at environmentally relevant levels, and ii) that early-life exposure windows may be more sensitive to antidepressant impact, leading to long-term effects on reproductive performance. One possible route for early exposure may be through maternal transfer of the compounds to the oocytes, especially given the bioaccumulation potential of the antidepressants in tissues including gonads (6, 59, 78, 80, 81). The mechanism(s) by which antidepressants may lead to changes in the developmental programming of the reproductive axis remains to be examined.

**Table 1:** Evidence of endocrine impacts of antidepressants in fish.

Drug	Species (sex)	Treatment	Effects	Reference
***Reproduction***				
Fluoxetine	Mosquitofish (*Gambusia affinis*; both sexes)	59 to 159 days post fertilization (juvenile to adult) to 71 µg/L [W.E]	Delayed maturation of sexual morphology	(53)
	Goldfish (*Carassius auratus*; females)	14-day adult exposure to 5 µg/g body weight [I.P]	Reduced circulating estradiol, and transcript levels of ERβ1 in the telencephalon and hypothalamus, and ERα, in the telencephalon	(54)
	Goldfish (males)	7-or 14- day adult exposure to 0.54 or 54 µg/L [W.E]	Reduced milt volume, increased plasma estradiol, and increased transcript abundance of testicular lhr, fshr, and cyp19a at 54 µg/L	(55)
	Zebrafish (*Danio rerio*; females)	Adult exposure to 32 µg/L for 7-days [W.E]	Reduced ovary Aromatase-A transcript abundance, reduced ovary estradiol and overall egg production	(56)
	Killifish (*Nothobranchius fuzeri;* females)	Life-long to 5 µg/L [W.E]	Increased fecundity	(57)
	Mosquitofish (males)	Adult exposure to 30 and 380 ng/L [W.E] for 30 days	Increased sperm production	(58)
	Japanese medaka (*Oryzias latipes;* females)	Adult 28-day exposure to 0.1 and 0.5 µg/L [W.E]	Increased estradiol levels in the plasma	(59)
Citalopram	Zebrafish (males)	1. Adult 14-day exposure to 4, 10, or 100 µg/L [W.E] 2. Adult 30-day exposure to 40 or 100 µg/L [W.E]	1. Reduced transcript levels of gnrh3 in the brain, fshβ in the pituitary, and lowered density of GnRH3 and serotonin fibers in the brain 2. Reduced transcript abundance of gnrh3 in the brain and fshβ in the pituitary and reduced spermatozoa, spermatogonium, and secondary spermatocytes	(60)
Venlafaxine	Fathead minnows (*Pimephales promelas*; both sexes)	Lifelong exposure to 1. 0.88 µg/L or 2. 88 µg/L [W.E]	1. Increased genital papillae and ovipositor area. 2. Increased egg production	(61)
	Zebrafish (females)	Adult 6-week exposure to 10 µg/L [W.E]	Reduction in total fecundity and alterations in kidney tubule morphology	(62)
	Zebrafish (females)	Adult 21-day exposure to 1 µg/L [W.E]	Changes in miR-22b, miR-301a miRNA in gonad	(63)
***Growth***				
Fluoxetine	Goldfish (female)	Adult 14-day adult exposure to 5 µg/g body weight [I.P]	Reduced growth rate	(64)
	Meagre (*Argyrosomus regius)*	Exposed to 3 µg/L for 15 days as juveniles	Reduced length, weight, and specific growth rate, and observed DNA damage in the liver	(65)
	Zebrafish	Exposed to 100 µg/L for 30 days as adults	Reduced pseudo specific weight gain and feeding	(66)
	Killifish (males and females)	Lifelong exposure to 5 µg/L	Reduced body length	(57)
Sertraline	Fathead minnow larvae	Exposed to 30, 60, 120, 250 µg/L for 48 h from ~48 hpf at pH 1. 6.5, 2. 7.5, and 3. 8.5	Growth retardation and reduced feeing responses	(67)
	Yellow catfish (*Pylodictis olivaris*)	Exposed to 1, 10 or 100 µg/L for either 7 or 14 d at the juvenile stage	All concentrations reduced weight gain and specific growth rate. Higher concentrations (10 and 100) reduced brain transcript abundances of sst, gh, and igf1, with all concentrations reducing npy	(68)
Venlafaxine	Fathead minnows	Life-long exposure to 88 µg/L	31 dpf juveniles exhibit a significant reduction in body weight	(69)
	Zebrafish	Exposed to 1 or 10 ng at the zygotic stage [M.I]	Increases developmental rate and length in larvae	(41)
	Zebrafish	Exposed to 1 or 10 ng at the zygotic stage [M.I]	Growth retardations at the juvenile stage (60 dpf). Increased hepatic somatic index, and decreased liver transcript abundances of GHrs, IGF2, and lepa, and reduced whole-body insulin.	(70)
	Zebrafish	Exposed to 1 or 10 ng at the zygotic stage [M.I]	Reduced serotonin in the gut of 48 hpf fish.	(39)
	Zebrafish	Exposed to 1 µg/L [W.E] from 2 hpf	Increased length, head area, and eye size at 72 hpf.	(71)
	Fathead minnows	Exposed to 0.06, 0.33, or 3 µg/L [W.E] from 2 hpf to 7 dpf	Reduced growth	(72)
***Stress response***				
Fluoxetine	Gulf Toadfish (*Opsanus beta)*	24 h exposure to 50 ug/g [I.P]	Increase in plasma cortisol levels	(73)
	Zebrafish larvae	Exposure to 0.54 and 54 µg/L [W.E] from 3 hpf to 144 hpf	Decreased unstressed and stressed cortisol levels at 96, 120, and 144 hpf	(74)
	Zebrafish males and females	Exposure to 0.54 and 54 µg/L [W.E] from 3 hpf to 144 hpf	Reduced whole-body cortisol in males, reduced cortisol in stressed females at the higher concentration. Disrupts genes associated with cortisol-related biological pathways in the head kidney of males.	(75)
	Zebrafish males and females	Exposure to 0.54 and 54 µg/L [W.E] from 1. 0 to 15 dpf, or 2. 15 to 42 dpf.	1. 0.54 µg/L reduces stressed cortisol levels in females, with both levels reducing cortisol production in stressed males. 2. 54 µg/L reduces cortisol in both stressed males and females.	(76)
Venlafaxine	Rainbow trout (*Oncorhynchus mykiss*)	Exposed to 1.0 µg/L for 7 days at the adult stage	Increased transcript abundance of CRF in the hypothalamus, and pomcb in the hindbrain	(36)
	Adult zebrafish females	Exposed to 1 or 10 ng at the zygotic stage [M.I]	Reduced cortisol levels in female following a stressor.	(77)

List of experiments sorted by endocrine disrupting effect and drug, with the species and sex impacted, along with the treatment protocol, noted effects, and the reference provided. Water exposures [W.E], intraperitoneal injection [I.P], and microinjection [M.I] studies are shown.

Although the mode of action of antidepressants in affecting reproductive endocrine disruption is unclear, one possible route may be through the modulation of the monoaminergic system. Indeed, the functioning of the hypothalamic-pituitary-gonadal (HPG) axis in fish (9, 50, 82, 83) is modulated by monoamines, including serotonin, norepinephrine, and dopamine (46, 47, 52). For example, dopamine plays an inhibitory role in fish reproduction by inhibiting gonadotropin releasing hormone (GnRH) from the hypothalamus and gonadotropins from the pituitary (48). Similarly, serotonin stimulation also regulates HPG axis (12), including stimulation of GnRH at the hypothalamus (84), and stimulation of oocyte maturation locally in the gonads by increased synthesis of estrogen and maturation-inducing steroids (85). The monoamines may also play differing roles at specific developmental stages or across species. For instance, injections of tyrosine and tryptophan (precursors of dopamine and serotonin, respectively) for 10 days in Gulf killifish (*Fundulus grandis*) increases gonadosomatic index in males (86). Conversely, in mummichog (*Fundulus heteroclitus*), serotonin exposure inhibits oocyte maturation during the follicular development stage *via* activation of the cAMPK-PKA transduction pathway (87, 88). Taken together, alterations in monoamine levels may be a possible mechanism by which antidepressants impact reproductive endocrine disruption, but these effects may be species-, life stage- and sex-dependent. Studies are clearly warranted with environmentally relevant species using environmentally relevant concentrations to understand the risks posed on the reproductive performance of fish.

### Growth

Growth in fish is regulated through a variety of physiological pathways mediating energy acquisition and balance (89, 90). The drive to feed is regulated by the neuropeptides (reviewed by 91), with the somatotropic axis playing a key role in modulating growth and metabolism of skeletal muscle, which comprises >50% of the fish mass (89). Environmental exposures to antidepressants have been shown to restrict growth in fishes, including goldfish (64), zebrafish (66, 70, 71), meager (*Argyrosomus* ost*regius*; 65), yellow catfish (*Tachysurus fulvidraco*; 68), short-lived killifish (57), and fathead minnows (67, 69, 72; See **Table 1** for specific experimental information). This is an important consideration, as restriction on growth is true of exposures to both the SSRI (fluoxetine, sertraline) and the SNRI class (venlafaxine), and has been noted even at environmentally relevant concentrations (68, 71, 72). Exposure during critical developmental windows appears to be more sensitive, as early-life (72) and zygotic (70) exposures led to growth retardations at later life-stages. These growth effects may be related to possible endocrine disruptions. A key aspect of growth is the endocrine control of feeding and metabolism (89, 90). Antidepressants exposure have been shown to reduce feeding responses in fish (92), including goldfish (64), stickleback *(Gasterosteus aculeatus*; 93), fathead minnows (94, 95), rainbow trout (36), hybrid striped bass (37, 38, 96), mosquito fish (79), and European perch *(Perca fluviatlis*; 92). As the drive to feed is regulated by the expression of neuropeptides (reviewed by 91), it is possible that these peptides are targets for the impact of the drug. For instance, antidepressants have been shown to influence several key genes involved in feeding activity in fish, including proopiomelanocortin b, corticotropin releasing hormone (CRH), and neuropeptide Y, and these changes correlated with reduced food intake (36, 64, 68), which may be reflected in reduced growth rates. The growth hormone (GH) and the insulin-like growth factors (IGFs), and their receptors and binding proteins are all key components of the somatotropic axis, orchestrating growth and metabolism in fish (97–99). The functioning of the somatotropic axis is regulated by monoaminergic stimulation in fish (10, 100). For instance, serotonin stimulation increases GH secretion (101), while catecholamines, including norepinephrine reduce GH secretion (102). Dopamine also pays a role in modulating GH release in fish (reviewed by 8). For instance, exposure to L-DOPA (precursor of dopamine), dopamine, or D_1_/D_2_ receptor agonists (apomorphine) by intraperitoneal injection increased the serum levels of GH in goldfish (102). This increase in GH appears to be driven primarily by the activation of the D_1_ receptor (103), and involves the cAMP-PKA pathway (104). We have also previously shown that venlafaxine reduced the transcript abundances of growth hormone receptors, IGF2, leptin a, the leptin receptor, and myostatin, in the liver of zebrafish, possibly signaling a disruption in the growth axis functioning, and also leading to metabolic disruption (70). A similar effect was also seen following sertraline exposure in the yellow catfish, experiencing reductions in somatostatin, growth hormone, igf, and neuropeptide y transcript abundance in the brain (68). Together, given the modulatory role of monoamines on the growth axis, it is likely that alterations to their levels due to antidepressant exposure has the potential to impact the feeding and growth performance of fish. Importantly, our study demonstrated that an early-life exposure to venlafaxine can lead to long-term perturbations in the programming of the growth axis (70), but whether this occurs at environmental levels of the antidepressant remain unexplored.

### Corticosteroid Stress Response

The endocrine stress response is highly conserved in vertebrates and is essential to allow animals to cope with stress (105–109). The primary stress response includes the sympathetic nervous system activation leading to the release of catecholamines, and this is followed by the release of the corticosteroids from the adrenal glands in mammals and the interrenal tissue in teleosts (105, 110–112). In fish, cortisol is the major corticosteroid released in response to stress and its release is mediated by the activation of the hypothalamus-pituitary-interrenal (HPI) axis (106, 107, 111, 113). Once released into the circulation, cortisol action in target tissues is mediated by the activation of either the glucocorticoid receptor (GR) and/or the mineralocorticoid receptor (MR; 114–116). Monoamines play an important role in the regulation of the corticosteroid stress axis (117, 118). In fish, studies have demonstrated that serotonin influences downstream cortisol production (119, 120). For example, intraperitoneal injection of 8-OH-DPAT, a 5HT_1A_ receptor agonist, elevates basal levels of cortisol, and inhibits the attendant rise in cortisol following an acute stressor in juvenile Arctic charr (*Salvelinus alpinus*; 121). This was also mimicked by increased intake of tryptophan, which counteracted the stress-induced elevations in plasma cortisol in rainbow trout (122). Chromaffin cells of the head kidney have been shown to contain serotonin, and this monoamine is capable of stimulating the release of cortisol from interrenal tissues *in vitro* (123). Catecholamines can also exert influence on the stress axis function, with noradrenaline capable of stimulating CRH secretion in tilapia *in vitro* (124), and the injection of noradrenaline and adrenaline stimulating the release of cortisol in sea bass (*Dicentrarchus labrax*; 125). Dopamine, in contrast has been shown to inhibit cortisol release (121), and this may occur even at the level of the pituitary (126). Consequently, the monoaminergic system exerts control over the activity of the HPI axis and may be a target for the antidepressant impact on the stress axis in fish.

Investigations of possible impacts to the stress axis in fish by antidepressants have primarily been carried out using fluoxetine and venlafaxine, with recent evidence suggesting impacts may be sex-specific (See **Table 1** for specific experimental information). The responses seen in the activity of the stress axis appears to be life stage-specific in fish. For instance, the circulating cortisol levels increase following an intraperitoneal administration of 50 µg/g of fluoxetine in the adult Gulf toadfish (*Opsanus beta*) (73). Also, juvenile rainbow trout exposed to environmental levels of venlafaxine (0.1 and 1 ug/L) display amplified cortisol responses following a social stressor, and this correlated with increases in the transcript abundance of *crh* and *pomcb* in the brain. Conversely, antidepressants exposure to early-life stages suppressed the stress-induced cortisol production, and also led to long-term impacts on cortisol response, suggesting alterations in the developmental programming of the stress axis (74, 75, 77). For instance, early life exposure to fluoxetine (either 0.54 µg/L or 54 µg/L) reduces basal and stressed levels of cortisol in zebrafish (75). When these fish grew to adults, the attenuated stressor-induced cortisol response was sexually dimorphic with the males more sensitive than females (75, 127). In contrast, zygotic deposition of venlafaxine did not influence cortisol levels at the larval stage, but the stressor-induced cortisol production was attenuated in the female and not male fish as adults (77). Vera-Chang and colleagues (75) suggested that disruptions at the level of the head kidney limited cortisol production in response to fluoxetine, while venlafaxine exposure revealed that the head kidney was still responsive to ACTH (75), suggesting that the disruption in cortisol production was at the level of the hypothalamus and/or pituitary. Although the route of exposure and duration of exposure were different between the two antidepressants (75, 77, 127), the results suggest that the mode of action in affecting the stress axis functioning may be distinct. At least in zebrafish, sex-specific differences in adrenoreceptors are seen in females relative to males, particularly in the hypothalamus and pre-optic areas, with female fish exhibiting higher levels of α_2_ adrenoreceptors (128). It remains to be seen if the difference in sex-specific effect seen due to early life exposure to either fluoxetine or venlafaxine may be related to their effect on the reuptake of serotonin or serotonin and norepinephrine, respectively. Taken together, these studies demonstrate that the class of antidepressants and duration of exposure may influence the outcome of the endocrine disruption of the stress axis in a sex-specific manner. Regardless, disruptions in cortisol levels may compromise their stress coping capability, as cortisol and the concomitant activation of GR and/or MR are integral players in the energy substrate partitioning to cope with stress (114–116, 129). The mechanisms leading to antidepressants effect on the stress axis warrant further study, especially given that some of these changes are even passed on to successive generations (63, 74, 75, 77, 127). This leads to the proposal that epigenetic changes due to early life exposure to the antidepressants may be involved in the long-term programming of the stress axis in teleosts.

## Conclusions and Future Directions

**Figure 1:**
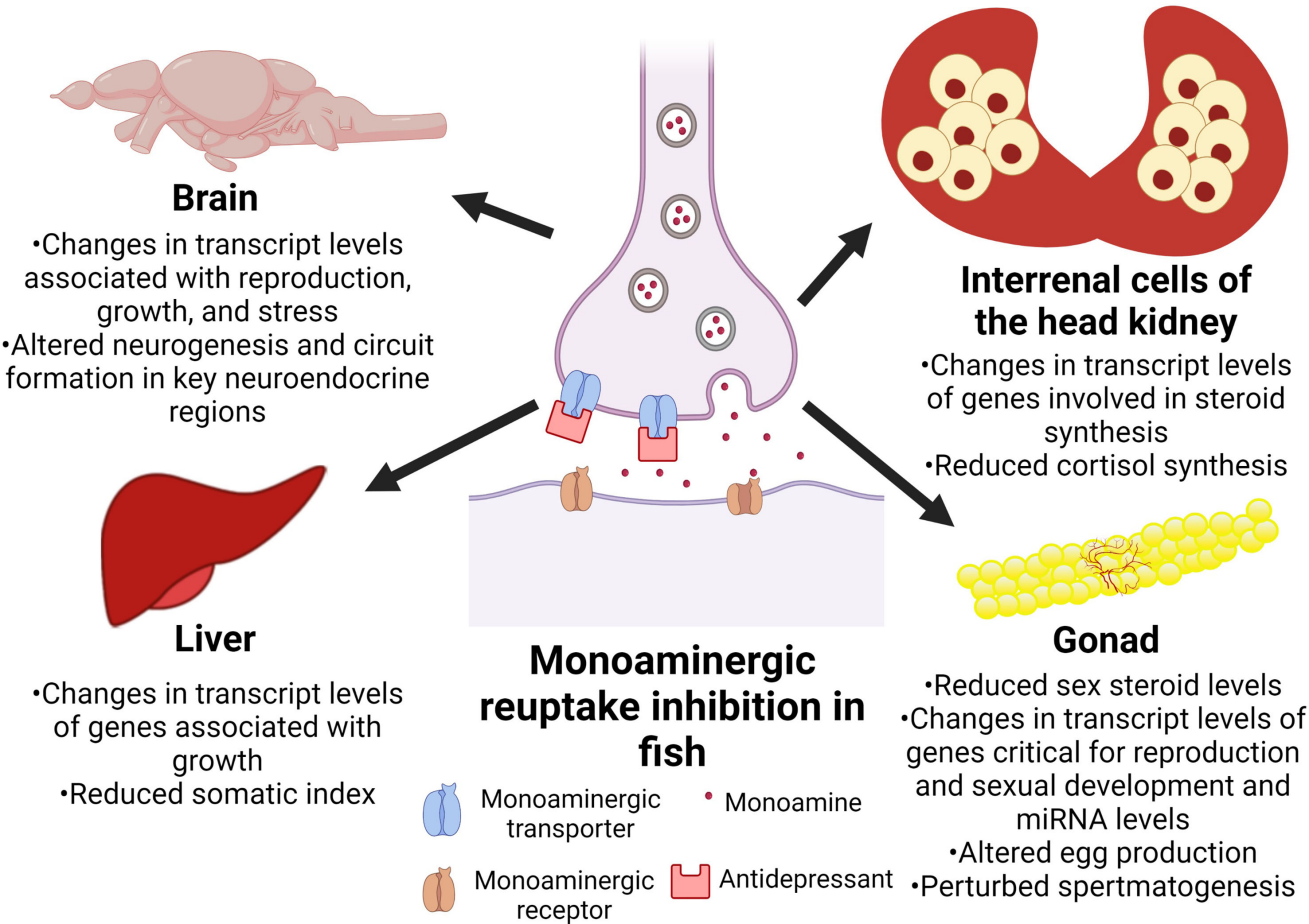
Overview of the tissue-specific impacts of antidepressants on the endocrine axes in fish. Depiction of the expected mode of action of antidepressants, along with the specific tissues and the observed phenotypes associated with exposures to antidepressants.

Antidepressants alter central monoamine levels, and this may play a role in the behavioural perturbations in fish (6, 39, 128). The emerging evidence also points to antidepressants as endocrine disrupting compounds in fish (**Table 1**). Specifically, studies reveal that the antidepressants impact the endocrine control of growth, stress, and reproductive axes in fish (**Figure 1**). As the monoaminergic system modulates endocrine function, a possible mode of action of the antidepressants in affecting endocrine disruption may be through alterations in the central and peripheral monoamine content, but this remains to be established. It is particularly important to note that early life exposures to antidepressants impacts the developmental programming of the endocrine system, and this may be reflected in long term and multigenerational changes in hormone levels and function (75, 77).We recently showed that venlafaxine-mediated disruption in embryonic serotonin content alters neurogenesis (39), and whether this alteration in brain development may play a role in the developmental programming of the endocrine axes warrants further study. For instance, the availability of knockout models in fish, including tryptophan hydroxylase paralogs (92, 129), a rate limiting enzyme in serotonin synthesis, will be an important step in delineating the effects of antidepressants mediated by the serotonergic pathway. While targeting the changes in the monoamine content may be one possible mechanism leading to endocrine disruption, the possibility that antidepressants may have direct effect on hormone action by targeting receptor function cannot be excluded (62). The sex of the animal is also another key factor that may dictate the outcome of the antidepressants impact (73, 75, 77, 127), and should be taken into account in future studies on endocrine disruption. Elucidating the mechanism of action of antidepressants in disrupting endocrine function in non-target organisms would be an important future direction to assess their impact on fitness consequences to the animal. A major limitation of the current body of work is the scarcity of studies on the endocrine effects related to environmentally relevant concentrations of the antidepressants in species commonly found in the aquatic habitats receiving MWWE. Environmental disruptors, including antidepressants are persistent in the aquatic habitats, suggestive of life-long exposures to these compounds (28). However, the implications of such life long exposures on animal fitness, and multigenerational consequences await further study. Such studies would be essential in developing markers for environmental risk assessment and for establishing adverse outcome pathways related to endocrine disruptive impacts of antidepressants. From a mechanistic standpoint, future studies should aim to identify the critical window during early development that is most sensitive to antidepressants and understand the mechanisms of action by which these pharmaceuticals alter the developmental programming of the endocrine system. This would allow for the development of novel biomarkers that are indicative of the developmental origin of endocrine dysfunction due to antidepressants exposure in fish.

## Author Contributions

All authors listed have made a substantial, direct, and intellectual contribution to the work and approved it for publication.

## Funding

The authors thank the funding support from the Natural Sciences and Engineering Research Council of Canada Discovery Grant awarded to MMV (RGPIN-2019-06291).

## Conflict of Interest

The authors declare that the research was conducted in the absence of any commercial or financial relationships that could be construed as a potential conflict of interest.

## Publisher’s Note

All claims expressed in this article are solely those of the authors and do not necessarily represent those of their affiliated organizations, or those of the publisher, the editors and the reviewers. Any product that may be evaluated in this article, or claim that may be made by its manufacturer, is not guaranteed or endorsed by the publisher.
